# Foundation of the International Collaborative of Surgical Education and Training (ICOSET)

**DOI:** 10.1111/ans.70866

**Published:** 2026-07-15

**Authors:** John P. Collins, Adrian Anthony

**Affiliations:** ^1^ Departments of Surgery and History University of Auckland Auckland New Zealand; ^2^ Department of Surgery University of Adelaide Adelaide Australia

Born of necessity and developed by consensus, the concept of an International Collaboration of Surgical Educators and Trainers (ICOSET) was first agreed at an inaugural conference in Melbourne in 2008. ICOSET was founded to provide a forum where surgical educators from around the globe could come together to share the issues they faced, discuss possible solutions and learn about new innovations in medical education. Transforming this aspiration into a reality required a formal structure and business plan, and a host organisation willing to underwrite these conferences. The inherent difficulties in achieving these requirements resulted in an ad hoc arrangement of finding organisations and convenors willing to organise the subsequent conferences, until a not‐for‐profit organisation partnered with the International Society of Surgery (ISS) was established in 2025. This article provides a brief history of the foundation of ICOSET and the subsequent challenges experienced in transforming the original concept into a sustainable reality.

Informal discussions between those responsible for the education and training of surgeons in different countries demonstrated the remarkably similar challenges being encountered. After decades of stability, an increasing number of factors began to affect the traditional methods used for training surgeons. These condensed into three main factors: generational challenges, regulatory and accountability requirements and competency‐based training. New directions of medical schools changed the demography of their student population and modified the content of the curriculum. The introduction of short‐stay and ambulatory care, and of minimally invasive operations, changed the practice of surgery. Safe working hours, an increasing regulatory environment and advances in educational methods including surgical skills laboratories began to question whether the traditional apprenticeship model for training surgeons remained appropriate or in need of major reform. In addition, an increasingly litigious world demanded more transparent and defensible methods for the selection of surgical residents and their subsequent assessment. It had also become clear that greater recognition and support were required to sustain appropriately equipped surgical teachers. Furthermore, surgeons voiced concerns that the existing conferences they had attended on medical education failed to cater for their specific needs. These factors were compounded by the simultaneous increase in public demands for greater accountability regarding the adequacy of surgical services being provided to the community.

At the commencement of the 21st century, the Royal Australasian College of Surgeons (RACS), which is responsible for the selection, training, assessment and certification of surgeons in Australia and New Zealand, began to experience unprecedented criticisms and negative public press relating to its educational practices and processes including selection. Allegations were initially raised in the Baume Report, about the ‘excessively tight control of the supply of trained surgeons by the Royal Australasian College of Surgeons’, [which] ‘will not supply … sufficient surgeons to meet reasonable standards of provision for Australia’ [1]. At the same time hospital administrators formally complained that RACS's selection processes restricted the number of surgical trainees necessary to safely staff their hospitals.

As a result, the Australian Competition and Consumer Commission (ACCC) which is ‘Australia's national competition, consumer, fair trading and product safety regulator’ [2], and its New Zealand equivalent, initiated investigations into RACS's selection and educational practices and processes in 2000. This was immediately followed by similar reviews undertaken by the Australian Medical Council and the Medical Council of New Zealand. Although no evidence was found to substantiate the original major criticisms, the external reviewers and members of the College Council and staff identified several opportunities for improvements [3]. Preparation for and participation in these very searching reviews, and in the implementation of the recommendations, highlighted the importance of being fully acquainted with how similar surgical educational organisations in other countries were responding to their own specific challenges.

During the same period, a crisis arose during the introduction of the Modernising Medical Careers programme in the United Kingdom. This programme was designed to reconfigure postgraduate medical education throughout the United Kingdom. However, it resulted in major criticisms, resignations, boycotts by senior medical practitioners, widespread protests by junior doctors and eventually a review by the House of Commons Health Committee [4].

In response to these local and international events, the concept arose of establishing an international collaboration of surgical educators, who could come together to share the issues they faced, and learn about possible solutions including new innovations in medical education. This idea was first canvassed with leaders of the Council of the Royal Australasian College of Surgeons, and later with representatives of various surgical colleges and similar organisations around the world. Encouraged by the responses received, an international foundation ‘faculty’ (Table 1) was established with the aim of convening an appropriate educational conference for surgeons. The ‘faculty’ also proposed that efforts should be made to try and reach international consensus statements on the areas of greatest controversy.

**TABLE 1:** Membership of the inaugural faculty of ICOSET.

John Collins—(Convenor), Royal Australasian College of Surgeons (RACS)
Richard Canter—Royal College of Surgeons of England (RCSEng)
Ian Civil—Royal Australasian College of Surgeons (RACS)
Timothy Flynn—American Board of Surgery (ABS)
Richard Reznick—Royal College of Physicians and Surgeons of Canada (RCPSC)
David Rowley—Royal College of Surgeons of Edinburgh (RCSEd)
William Thomas—Royal College of Surgeons of London (RCSEng)
Oscar Traynor—Royal College of Surgeons in Ireland (RCSI)

After obtaining external funding and with the full support of the College President Dr Andrew Sutherland, the inaugural conference took place over 2 days in March 2008 at the Royal Australasian College of Surgeons in Melbourne. Because it was unlikely that a new standalone conference on surgical education and training would be viable, this was co‐badged with the 13th Ottawa International Conference on Clinical Competence held in Melbourne during March 2008. It also enabled the organisers of both conferences to share speakers. Invitations were sent to various surgical institutions around the globe. One hundred and ninety delegates from thirteen countries participated in the conference on surgical education. Twenty‐three invited speakers addressed major topics during six plenary sessions. A consensus was reached on 10 principles of selection (Table 2) and later published as *The Melbourne International Consensus on Selection of Surgical Trainees* [5]. It was not possible to reach a consensus on selection methodology. Conference proceedings comprising 26 articles by the leading participants were published in a Special Issue of the *Australia and New Zealand Journal of Surgery* [6]. At the conclusion of the conference, delegates voted unanimously to support the proposal that an International Collaboration of Surgical Educators and Trainers (ICOSET) be founded, and a biennial international conference on surgical education and training take place, rotating between different international surgical institutions or colleges.

**TABLE 2:** Ten principles on selection (material from Collins et al. 2019).

Melbourne international consensus statement on selection
Responsibility for selection must involve trained members of the surgical profession and the agencies (including employers) responsible for the delivery of education and training
Selection must aim to identify those doctors with the values, attitudes and aptitude required to become competent surgeons
Eligibility criteria (long‐listing) for application to specialist surgical education and training should include generic and specialty‐specific components
Selection methodology must be predetermined and transparent, include a broad range of approaches to maximise validity and reliability, involve multiple raters, contain clear criteria for marking and allocate weighting for each tool which permits ranking of applicants.
Potential for successful training in a speciality programme is the basis for selection and not the extent of prior knowledge, experience and skills in that specialty.
Structured curriculum vitae provide important verifiable biographical information on clinical experience and academic and other accomplishments.
Structured referees' reports can provide credible information from surgeons, colleagues, other healthcare professionals and employers based on their first‐hand experience of a doctor's performance in the working and learning environment.
Structured interviews should use questions which target specific competencies identified through job analysis and yield important information not available from other selection tools.
Knowledge is an essential base for clinical reasoning and judgement. The extent of a candidate's knowledge at the extremes of performance is a good predictor of their future overall performance.
Early selection into a surgical education and training programme must be accompanied by clearly established grounds and methodology to ensure struggling or underperforming trainees do not progress unless competency deficiencies are rectified.

*Note:* Authors: John Collins, RACS Australasia; Richard Carter, RCSEng; Ian Civil, RACS NZ; Timothy Flynn, ACS; Richard Reznick, RCPSC; David Rowley, RCSEd; William Thomas, RCSEng and Oscar Traynor, RCSI.

However, the inherent challenges of transforming this decision into a reality soon became obvious. Initial discussions identified the need for a formal structure and business plan, and a host organisation willing to underwrite these conferences. Faced with these difficulties, it was decided in the interim to continue with an ad hoc arrangement of finding host institutions and convenors willing to organise the conferences until a more formal partner association could be secured.

Following the inaugural ICOSET conference in Australia in 2008, a further nine meetings have taken place in seven different countries (Table 3). The second ICOSET conference was hosted by the Royal College of Surgeons in Ireland and convened by Professor Oscar Traynor. The William Halsted Lecture was first introduced at this meeting. Two hundred and fifty delegates from 22 countries participated in this conference, confirming the importance of its international role in the education and training of surgeons. Although an international consensus statement was eventually reached on the training of surgeons in an era of reduced working hours [7], many issues remained unresolved. While the founding faculty continued to maintain the importance of reaching consensus statements on the most challenging areas in surgical education and training [8], it eventually became too difficult to achieve. The diverse backgrounds of delegates and the significant variations between health care delivery and training environments in the different countries made reaching consensus statements very challenging. Furthermore, the robust and intractable views of some delegates were not conducive to the collegial environment envisaged by the founding faculty. It was, therefore, reluctantly decided to abandon this important aim of ICOSET, at least for the time being.

**TABLE 3:** ICOSET conferences and key themes.

Year	City	Host organisation	Convenor	Key themes
2008	Melbourne	RACS	John Collins	Trainee selection, simulation in training, assessment and feedback, supervision and mentorship
2010	Dublin	RCSI	Oscar Traynor	Impact of reduced work hours, role of simulation, impact of technology on training, patient safety
2012	Ottawa	RCPSC	Kenneth Harris and Richard Reznick	Training for society needs, impact of simulation, non‐technical skills, competency‐based training
2014	Harrogate	RCSEng	Jonathan Beard	Curriculum design, surgeons as educators, integrating technology, non‐technical skills, quality assurance
2015	Copenhagen	CAMES	Lars Konge	Competency‐based training, culture of training, training pipeline, use of IT in training
2017	Adelaide	RACS	Stephen Tobin	Workforce needs, work‐based assessments, faculty development, non‐technical skills, cultural competency
2019	Edinburgh	RCSEd	Craig McIlhenny	Professionalising surgical education, programmatic assessment, educational design, diversity in training
2022	Edinburgh	RCSEd	David O'Regan	Disrupted training, refining assessment, hybrid educational frameworks, trainee wellbeing, sustainability
2025	Edinburgh	RCSEd	Alexander Phillips	Ai in surgical education, cross‐industry insights, training for leadership, resilience and wellbeing, simulation
2026	Mexico City	ISS	David O'Regan	Organisational models in surgical education, best‐practice approaches, contextualised education design, training standards and standardisation, simulation, global perspectives on surgical training

Meetings continued biennially as a 2‐day programme and a prelude to other surgical conferences to optimise visibility and accessibility. Each conference was themed to address issues contemporary at the time (Table 3). Programmes featured leaders in surgical education, surgeons, medical educators, content experts and trainees. Each conference adopted an evidence‐based focus and sought to deliver pragmatic outcomes through an interactive format of debates, workshops, masterclasses, plenary sessions and poster discussions. By 2018, the Royal College of Surgeons of Edinburgh, through its Faculty of Surgical Trainers, had assumed governance responsibility for future conferences. This governance model, however, was not conducive to ICOSET evolving with a truly global projection. Surgical education from diverse geopolitical, socio‐economic and cultural parts of the world was unrepresented. ICOSET was at risk of blinding itself to the broadest possible perspectives on surgical education. The opportunity to adopt a global lens, untethered to surgical colleges, surgical specialty societies or nations, was taken in July 2025 by transforming ICOSET from a Conference to ICOSET as a registered, not‐for‐profit, independent Collaborative. The organisation formed a Board of Directors with fiduciary responsibilities and terms of reference. Its vision is to advance and professionalise surgical education for optimal patient and community outcomes. Its mission is to foster global collectiveness in collaborating, supporting and sharing practice, research and innovation in surgical education.

In a milestone development, ICOSET was accepted as a participating society during the biennial International Surgical Week 2026 programme in Mexico City, convened by the International Surgical Society (ISS) [9]. At its first international outing as an organisation, ICOSET's sessions on surgical education brought a new dimension to the ISW programme. The programme highlighted a critical gap in global surgical discourse on the inherent role surgical education plays across the spectrum of surgical care and in the ability of surgeons to serve the needs of their communities. Recognising that ICOSET is well placed to inform this discourse, ISS will be represented on the Board of ICOSET via its Director of Surgical Education. There appears to be natural alignment of ICOSET's vision and mission with the strategic goals of ISS. Over the next years, ICOSET aims to develop and operationalise an educational strategy that spans technical and non‐technical domains and to promote the sharing of best‐practice surgical education, contextualised to where surgical care is delivered. This ensures surgical education remains inherent to the social contract that surgery has with society, no matter the country, culture or generation. ICOSET will engage medical students and trainees in its governance model and extend collaboration with stakeholders to enact its mission. And it will convene a surgical education programme at the next ISW in Cape Town in 2028 to continue the socialisation of surgical education on a global footing. The journey of ICOSET continues the importance of global collaboration in advancing the training of surgeons and consequently, better outcomes for patients.

## Author Contributions

**John P. Collins:** conceptualization, writing – original draft, writing – review and editing. **Adrian Anthony:** writing – review and editing, writing – original draft.

## Data Availability

The data that support the findings of this study are openly available in University of Auckland at https://www.auckland.ac.nz.
